# A pharmacokinetic-pharmacodynamic assessment of oral antibiotics for pyelonephritis

**DOI:** 10.1007/s10096-019-03679-9

**Published:** 2019-09-07

**Authors:** J. W. S. Cattrall, E. Asín-Prieto, J. Freeman, I. F. Trocóniz, A. Kirby

**Affiliations:** 1 University of Leeds, Leeds, LS2 9JT UK; 2 Pharmacometrics & Systems Pharmacology Research Unit, Department of Pharmaceutical Technology and Chemistry, School of Pharmacy and Nutrition, University of Navarra, Pamplona, Spain; 3 IdiSNA, Navarra Institute for Health Research, Pamplona, Spain; 4 Leeds Teaching Hospitals NHS Trust, Leeds, LS9 7TF UK; 5 Department of Microbiology, Old Medical School, Leeds General Infirmary, Leeds, LS1 3EX UK

**Keywords:** Administration, oral, Anti-bacterial agents, Pharmacokinetics, Pharmacodynamics, Modelling, Simulation

## Abstract

Antibiotic resistance to oral antibiotics recommended for pyelonephritis is increasing. The objective was to determine if there is a pharmacological basis to consider alternative treatments/novel dosing regimens for the oral treatment of pyelonephritis. A systematic review identified pharmacokinetic models of suitable quality for a selection of antibiotics with activity against *Escherichia coli*. MIC data was obtained for a population of *E. coli* isolates derived from patients with pyelonephritis. Pharmacokinetic/pharmacodynamic (PK/PD) simulations determined probability of target attainment (PTA) and cumulative fraction response (CFR) values for sub-populations of the *E. coli* population at varying doses. There are limited high-quality models available for the agents investigated. Pharmacokinetic models of sufficient quality for simulation were identified for amoxicillin, amoxicillin-clavulanic acid, cephalexin, ciprofloxacin, and fosfomycin trometamol. These antibiotics were predicted to have PTAs ≥ 0.85 at or below standard doses for the tested *E. coli* population including cephalexin 1500 mg 8 hourly for 22% of the population (MIC ≤ 4 mg/L) and ciprofloxacin 100 mg 12 hourly for 71% of the population (MIC ≤ 0.06 mg/L). For EUCAST-susceptible *E. coli* isolates, doses achieving CFRs ≥ 0.9 included amoxicillin 2500 mg 8 hourly, cephalexin 4000 mg 6 hourly, ciprofloxacin 200 mg 12 hourly, and 3000 mg of fosfomycin 24 hourly. Limitations in the PK data support carrying out additional PK studies in populations of interest. Oral antibiotics including amoxicillin, amoxicillin-clavulanic acid, and cephalexin have potential to be effective for a proportion of patients with pyelonephritis. Ciprofloxacin may be effective at lower doses than currently prescribed.

**Electronic supplementary material:**

The online version of this article (10.1007/s10096-019-03679-9) contains supplementary material, which is available to authorized users.

## Introduction

Pyelonephritis is a bacterial infection of the renal pelvis and kidney. It is a life-threatening infection that can lead to renal scarring and impairment of kidney function [1]. However, with adequate treatment, the infection can be cured without complications. The incidence of pyelonephritis varies depending on sex and age [1]. Estimates of outpatient pyelonephritis rates in females are 12–13 cases per 10,000 population annually [1]. The predominant aetiological agent of pyelonephritis is *Escherichia coli* in up to 84% of cases [1]. International guidelines (IDSA, ESCMID) recommend outpatient management of pyelonephritis with oral ciprofloxacin, levofloxacin, or oral trimethoprim-sulfamethoxazole [2]. However, antibiotic resistance to these antibiotics within populations of *E. coli* is increasing and complicates treatment for pyelonephritis [2]. Unfortunately, despite a wide range of alternative oral antibiotics having activity against *E. coli*, the use of orally available cephalexin, fosfomycin, mecillinam, nitrofurantoin, and trimethoprim at standard doses is excluded by EUCAST *E. coli* breakpoints [3]. There is therefore a limited range of recommended antibiotics for the oral treatment of pyelonephritis. If this range of antibiotics could be increased, it would be of benefit to patients, who could avoid the need for intravenous therapy, and associated costs and hospitalisations would be reduced. An initial step in considering if the range of antibiotics could be increased is an analysis of published antibiotic pharmacological data, including a pharmacokinetic analysis.

Understanding variability in antibiotic pharmacokinetics has relevant practical applications [4]. Given a schema of administration of antibiotic therapy, patient characteristics which impact on antibiotic pharmacokinetics, and bacterial susceptibility to an antibiotic, it is possible to make predictions about an antibiotic’s clinical efficacy. Alternatively, using a pharmacodynamic clinical efficacy target, patient characteristics which impact on pharmacokinetics and bacterial antibiotic susceptibility, it is possible to individualise antibiotic dosing regimens. In addition, if geographically restricted antibiotic susceptibility is used, it is possible to make predictions about antibiotic efficacy in specific geographical locations. Population pharmacokinetic/pharmacodynamic (PK/PD) modelling and simulation are recommended as a supportive approach to individualise therapy as it allows patient characteristics to be included as covariates of the PK/PD parameters of developed models [4].

## Objective

Our overall aim was to determine if there was a PK/PD basis which would support further investigation of alternative (non-guideline recommended) antibiotic regimens and/or MIC-based dosing regimens for the oral treatment of pyelonephritis. In order to accomplish this, we established the following objectives: (i) use of a model quality assessment methodology to select a pharmacokinetic model for assessed antibiotics, (ii) PK/PD analysis to elucidate the likelihood of success of the maximum standard antibiotic dose for the treatment of pyelonephritis caused by *E. coli*, and (iii) establish the minimum dose of antibiotic required for successful antibiotic therapy against defined populations of *E. coli*. In addition, we aimed to demonstrate how antibiotic pharmacological data could be used to allow recommended antibiotic dosing regimens to be produced for specific patient populations.

## Materials and methods

We searched for population models for a representative selection of antibiotics and used these models, in combination with *E. coli* MIC data, to perform PK/PD simulations. These simulations predicted the (i) efficacy of selected antibiotics at standard doses and (ii) minimum effective dose.

### Selection of pharmacokinetic models

PK models were identified through a systematic review of the literature, with a focus on population PK (popPK) models. This process included a search of major databases “Medline” and “EMBASE + Embase Classic” for relevant studies, followed by secondary reference searching and manual citation tracking for those antibiotics for which limited studies were identified. The antibiotics selected for the search were chosen to represent multiple antibiotic classes with activity against the target pathogen and included beta-lactams (amoxicillin, amoxicillin and clavulanic acid, cephalexin, mecillinam), fluoroquinolones (ciprofloxacin, norfloxacin), fosfomycin, nitrofurantoin, trimethoprim, and trimethoprim-sulfamethoxazole. Full details of the systematic review are provided in the Supplementary material including Tables A1–3. Whilst tissue antibiotic concentrations are relevant to cure, we focused on analyses performed with serum concentration data, as previously recommended, being a surrogate for tissue concentrations [5]. Urine concentrations were not considered, as pyelonephritis is an infection of the renal tissue, as opposed to the urinary collecting system.

### Pharmacokinetic model quality assessment

Assessment of PK model quality occurred in two stages. Firstly, the model building process was assessed using a “confidence in quality” (CIQ) score focused on diagnostic model checks including (i) simulation-based model diagnostics (SBMD), (ii) evaluation of the precision of model parameters, and (iii) goodness of fit (GOF) plots. Models required at least one diagnostic check to be considered for inclusion. Subsequently, where multiple models were available, a qualitative assessment of each model was performed to select one candidate for analysis. These assessments considered (i) how relevant study populations were to patients with acute pyelonephritis, (ii) quality of raw data used to develop the model, and (iii) the choice and use of diagnostic model checks (see Supplementary material). The selected studies for the included antibiotics are listed along with the population characteristics and study design information in Table 1.

**Table 1:** Summary data of the PK model data used in PK/PD simulations

Antibiotic	Study	No. of patients/setting/administration route/sample analysis method	Dosing and collection of blood samples: timing/total samples analysed	Patient characteristics	Summary of population description	Diagnostic model checks
Amoxicillin, Amoxicillin-clavulanic acid	De Velde [6] (2016)	28	Dosing: 875/125 mg, or 500/125 mg of a single dose. Collection: pre administration and at 0.5, 1, 1.5, 2, 2.5, 3, 4, 6, 8, 10, and 12 h post administration (10 and 12 h for 875 dosing only) Total samples: 1428	Age (years): 33 ± 7^a^	Male volunteers Aged between 18 and 50 years Good general health. Exclusion criteria: > 20% deviation from ideal weight for height	NMRSE^h^: N
		Setting: healthy volunteers		Male: 28/28		Bootstrap: Y
		Administration: oral		Weight (kg): 77 ± 8^a^		GOF^i^: Y
		ASTED^c^/HPLC^d^				SBMD^j^: Y
Cephalexin	Greene [7](1972)	5	Dosing: 500 mg intravenously as a single dose. Collection: during a 4 h period post administration. Total samples: not provided	Age (years): unknown	Healthy volunteers	NMRSE^h^: N
		Setting: unknown		Height (cm): unknown		Bootstrap: N
		Administration: IV^e^		Weight (kg): unknown		GOF^i^: Y
		Disc diffusion assay				SBMD^j^: N
Ciprofloxacin	Conil [8] (2008)	102	Dosing: 400 mg 12 hourly intravenously. Collection: post infusion and at various times over a 24 h period. Total samples: 588	Age (years): 60 ± 17^a^	Antibiotics were prescribed for sepsis Exclusion criteria: haemodynamically unstable	NMRSE^h^: Y
		Setting: ICU^f^		Male: 75/102		Bootstrap: N
		Administration: IV^e^		Weight (kg): 77 ± 16^a^		GOF^i^: N
		HPLC^d^		CRCL^F^ (mL/min): 89 ± 54^a^		SBMD^j^: Y
Fosfomycin	Parker [9] (2015)	12	Dosing: 4 or 6 g 6 to 8 hourly most commonly. Collection: pre administration and at 30 min, 45 min, 1 h, 1.5 h, 2 h, 4 h, and 6 h post administration. Where possible, sampling occurred during the first dosing interval and/or on days 2, 4, 5, 6, and 7. Total samples: 515	Age (years): 62.5 (57.8 to 75.0)^b^	Critically ill patients Exclusion criteria: age < 18 years	NMRSE^h^: N
		Setting: ICU		Male: 8/12		Bootstrap: Y
		Administration: IV^e^		Weight (kg): 72 (70 to 80)^b^		GOF^i^: Y
		HPLC^d^		CRCL (mL/min): 59 (52 to 99)^g^		SBMD^j^: Y

^a^(mean ± SD), ^b^ (median (IQR)). ^c^*ASTED*, automated sequential trace enrichment of dialysates; ^d^*HPLC*, high-performance liquid chromatography; ^e^*IV*, intravenous; ^f^*ICU*, intensive care unit; ^g^*CRCL*, creatinine clearance (Cockcroft-Gault); ^h^*NMRSE*, NONMEM relative standard errors; ^i^*GOF*, goodness-of-fit plots; ^j^*SBMD*, simulation-based model diagnostics; *Y*, yes; *N*, No. Data on all PK studies identified is presented in the Online resource Tables A2–A10

### Bacterial isolates and MIC testing

*E. coli* bacteraemia isolates were collected from Leeds Teaching Hospitals NHS Trust, UK, to provide contemporary and geographically restricted MIC data. All isolates were collected consecutively from patients clinically assessed as having had pyelonephritis in 2016. MICs were generated for each antibiotic using an agar incorporation method according to CLSI susceptibility testing guidelines; however, in testing amoxicillin-clavulanic acid susceptibility, a fixed concentration of potassium clavulanate (2 mg/L) was used as per EUCAST recommendations [3, 10]. Control organisms were *E. coli* ATCC 25922 and *E. coli* ATCC 35218 (β-lactamase producing strain). At least 100 isolates were tested for each antibiotic, with this being a pragmatically chosen sample size. Amoxicillin MIC summary results outside of the tested concentration range (> 128 mg/L) were assigned to an MIC value of 256 mg/L.

### Pharmacokinetic-pharmacodynamic simulations

#### Software

The R package mlxR (version 3.1.0) was used to conduct the analyses. It contains a set of functions that allows, through stochastic simulations, generation of pharmacokinetic profiles for a population of virtual patients, from which user required PK metrics such as peak and trough (*C*_peak_, *C*_min_, respectively) concentrations, time to reach the peak concentration (*T*_peak_), and the area under the curve of the drug concentration over time (AUC) to be calculated [11–13].

#### Conducting simulations

Pharmacometric simulations were performed using selected models. One thousand virtual patients were simulated by randomly varying individual PK parameter values using a log-normal distribution and the reported mean and inter-individual variability (IIV) from the literature (Table 2). When the IIV was not reported, the standard deviation of the individual parameter distribution was used. Concentration-time profiles were simulated over 24 h (time step = 0.01 h). For this purpose, selected PK models were computationally implemented using MlxTran language. Several models had to be adapted from intravenous administration to simulate single-multiple oral administrations of the antibiotic (cephalexin, ciprofloxacin, fosfomycin). As required, absorption constants, bioavailability, and protein binding data of the antibiotics were obtained preferably from selected references; otherwise, EUCAST rationale documents and/or other literature were consulted [14]. Inter-occasion variability was not considered in the selected models. Covariates present in pharmacokinetic models were included in all PK/PD analyses using average population values. Dose levels but not dosing intervals were evaluated.

**Table 2:** Pharmacokinetic and pharmacodynamic information used for PK/PD simulations

Antibiotic	Model reference	PD target	Bioavailability	Protein binding	Parameter values Typical (variance to two decimal places)*		Covariates included Equation for inclusion Values simulated
Amoxicillin, Amoxicillin-clavulanic acid	De Velde [6]	32.5% *f*T > MIC [14]	70% [15]	20% [15–17]	*N* = 4.41 (1.28) MTT = 0.524 h (0.22) Vm = 1220 mg/h (0.10) Km = 287 mg (0.97)	Vc = 27.7 L (0.12) Vp = 3.02 L CL = 21.3 L/h (0.07) *Q* = 1.7 L/h	Not applicable
Cephalexin	Greene [7]	40% *f*T > MIC [18]	95% [19]	12.4% [20]	Ka = 1.90 (0.68) h^−1^ [21] ^a^ K_12_ = 1.27 h^−1^ (0.13) K_21_ = 2.68 h^−1^ (0.22)	Ke = 1.62 h^−1^ (0.14) Vc = 10.9 L (0.80) Vp = 19.6 L (1.10)	Not applicable
Ciprofloxacin	Khachman [22]	90 = *f*AUC/MIC [23]	69% [24]	25% [14]	Ka = 2.7 h^−1^ [25, 26] Vc = 38 L (0.40) Vp = 73 L	CL = 19.61 L/h (0.18) Q = 60 L/h (0.52)	Creatinine clearance CL = θ1*(CRCL/91.7)^θ2 CRCL = 112.5 g θ1^d^ = 18 θ2^e^ = 0.42
Fosfomycin	Parker [9]	43 = *f*AUC/MIC [27]	37.5% [28]	Negligible [14]	Ka = 0.1 h^−1^ [29] Vc = 26.5 L (0.15) Vp = 22.3 L	CL = 4.99 L/h (0.84) ^a^ Q = 19.8 L/h	Creatinine clearance TVCL^c^ = CL*(CRCL/90) CRCL^g^ = 112.5 g CL = 4.996
							Body weight TVV^b^ = V*((WGT/70)^(0.75)) WGT^f^ = 75 g V^h^ = 26.5

*If variance is not included, then the typical values were used for simulations. % *f*T > MIC, % of time free drug is above the MIC; *f*AUC/MIC, the ratio of free drug under the curve/the MIC value; *MTT*, mean transit time of absorption; *N*, number of absorption transit compartments; *Vm*, maximal absorption rate; *Km*, amount corresponding to 50% *Vm*; *V*, *Vc*, *Vp*, apparent volumes of distribution of the central and peripheral compartments, respectively; *CL*, total clearance; *Q*, inter-compartmental clearance; *Ka*, first order rate constant of absorption; *Ke*, first order rate constant of elimination; K_12_, K_21_, first order rate constants of distribution between the central and peripheral compartments. ^a^mean value of days 2–7, ^b^TVV = typical value of the volume of the central compartment, TVCL^c^ = typical value of clearance, θ1^d^ = ciprofloxacin clearance value in the population for a mean CRCL Cockcroft of 91.7 mL/min, θ2^e^ = exponent representing the magnitude of change of ciprofloxacin clearance dependent on the patient creatinine clearance, WGT^f^ = body weight, CRCL^g^ = creatinine clearance, V^h^ = volume of distribution of the central compartment. Values selected for co-variate analysis chosen to represent a typical patient

#### Simulation outcome measures

Probability of target attainment (PTA) and cumulative fraction of response (CFR) were the key outcome measures. PTA is defined as the probability that a specific value of a pharmacodynamic index (i.e. quantitative relationship between a pharmacokinetic metric and a microbiological value) is achieved at a certain (minimum inhibitory) concentration; and CFR is defined as the expected population PTA for a specific drug dose and a specific population of microorganisms [30].

The PTA was used to determine minimum doses achieving PTAs of 0.9 (cut-off for treatment success) at various doubling MIC values in the range of 0.002–256 mg/L [31]. CFR values at maximum standard doses, according to the British National Formulary (BNF), of each antibiotic were determined for populations derived from the Leeds bacteraemia isolates (≤ MIC_50_, ≤ MIC_90_, the entire population, and at MICs ≤ EUCAST susceptibility breakpoint) (the MIC_90_ and MIC_50_ values were defined as the lowest concentration of the antibiotic at which 90 and 50% of the isolates were inhibited, respectively) [32]. In addition, simulations were also conducted to identify the lowest dose for which the CFR was above 0.9, considering the same bacterial populations. The PK/PD indices and pharmacodynamic targets (PDTs) used for each antibiotic simulation were selected from EUCAST rationale documents if available/valid; otherwise, from other publications, see Table 2 [14].

## Results

### Selection of PK models

Population PK models were selected for five of the ten antibiotics considered in the initial protocol: amoxicillin, amoxicillin and clavulanic acid, cephalexin, ciprofloxacin, and fosfomycin trometamol. For the remaining antibiotics (mecillinam, nitrofurantoin, norfloxacin, trimethoprim, and trimethoprim-sulfamethoxazole), no population PK model was selected to progress to PK/PD simulations due to an absence of models with any diagnostic model check. Table 1 provides a summary description of each selected study including the population characteristics and study design. The full details of the systematic review including the identified studies for all considered antibiotics are available in the Supplementary material including Tables A1–3. The PK models selected and the PK/PD index and PDT extracted and used for PK/PD analyses are listed in Table 2. The quality of the included models was limited in comparison with the desired model characteristics, see Supplementary material Table A9. No selected models were developed using data from patients with pyelonephritis, and only the model for ciprofloxacin had more than the recommended 60 patients included [33]. Additionally, except for amoxicillin and amoxicillin-clavulanic acid, models were developed after intravenous administration and therefore, absorption characteristics (bioavailability and rate constant) were retrieved from other references.

### MIC testing

The MIC_50_, MIC_90_, and the geometric mean of the MIC distribution for 106–108 *E. coli* isolates from Leeds during 2016 are presented in Table 3, along with the corresponding MIC breakpoints by EUCAST for *Enterobacteriaceae*. The MIC_90_ values were above the MIC breakpoint classifying *Enterobacteriaceae* resistance by EUCAST for amoxicillin, amoxicillin-clavulanic acid, cephalexin, and ciprofloxacin but not fosfomycin (see Table 3).

**Table 3:** MIC50, MIC90, and MIC geometric means for 106–108 *E. coli* bacteraemia isolates from Leeds in 2016

Antibiotic	Leeds *E. coli* population MIC data (mg/L)			EUCAST MIC Breakpoints (mg/L)		% of Leeds isolates resistant
	MIC_50_	MIC_90_	MIC geometric	Resistant	Susceptible	
Amoxicillin	> 128^a^	> 128^a^	45.1	> 8	≤ 8	57%
Amoxicillin-clavulanic acid	8	64	7.25	> 8	≤ 8	38%
Cephalexin	8	16	12.2	> 16^b^	≤ 16^b^	17%
Ciprofloxacin	0.03	64	0.08	> 0.25	≤ 0.25	20%
Fosfomycin	0.5	2	0.64	> 32^b^	≤ 32^b^	1%

^a^Where concentrations are displayed as greater than a value, the MIC was found to be outside the tested concentration range. ^b^Lower UTI EUCAST breakpoint

### Pharmacokinetic/pharmacodynamic simulations

**Fig. 1:**
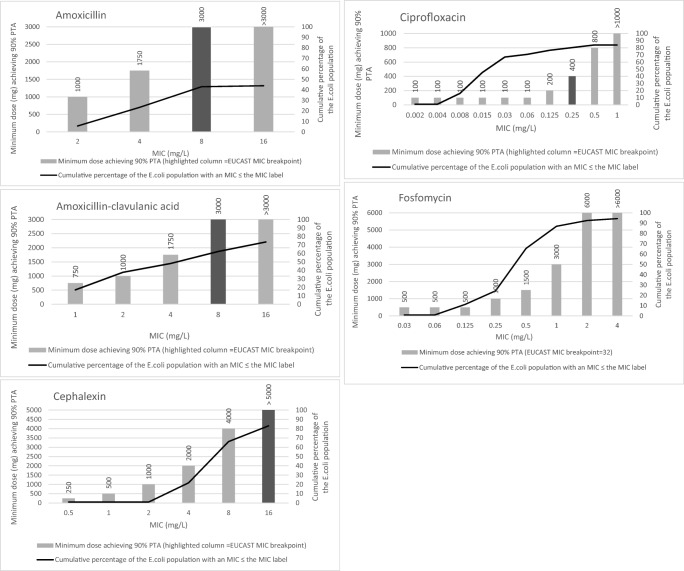
Minimum antibiotic doses able to achieve PTAs of ≥ 0.9 at various MIC values (highlighted columns being the EUCAST MIC breakpoint)

The results of the simulations are provided in Tables 4 and 5. Table 4 provides CFR data at the highest available standard doses. Table 5 provides the lowest doses for which a CFR ≥ 0.9 was achieved. The Supplementary material, Tables A4–A8, provides further detail on the individual PTA and CFR predictions for various combinations of MICs and doses. Figure 1 provides information on the minimum dose of antibiotic providing a PTA ≥ 0.9, along with the cumulative probability distribution of *E. coli* MIC data from Leeds isolates during 2016. As expected, when bacterial susceptibility decreases, higher doses are required to achieve the same probabilities of treatment success. Simulation results for each evaluated antibiotic are now described.

**Table 4:** Cumulative fraction of responses (CFRs) at maximum British National Formulary doses for various populations of the Leeds *E. coli* bacteraemia isolates

Antibiotic, dose, frequency	CFR for the *E. coli* population defined by their MIC values			
	≤ MIC_50_	≤ MIC_90_	Whole population	≤ EUCAST susceptible [3]
Amoxicillin 1000 mg, every 8 h	0.14	0.14	0.14	0.32 (8 mg/L)
Amoxicillin-clavulanic acid 500/125 mg, every 8 h	0.37	0.26	0.23	0.37 (8 mg/L)
Cephalexin 1500 mg, every 6 h	0.68	0.60	0.50	0.60 (16 mg/L)^a^
Ciprofloxacin 750 mg, every 12 h	1.00	0.90	0.84	1.00 (0.25 mg/L)
Fosfomycin 3000 mg, every 24 h	0.98	0.96	0.896	0.90 (32 mg/L)^a^

^a^Lower UTI EUCAST breakpoint

**Table 5:** Lowest dose of antibiotic achieving 90% CFR for specific populations of the Leeds *E. coli* bacteraemia isolates

	CFR for the population of MIC values							
	≤ MIC_50_		≤ MIC_90_		Whole population		≤ EUCAST susceptible [3]	
Antibiotics, frequency	Dose (mg)	CFR	Dose (mg)	CFR	Dose (mg)	CFR	Dose (mg)	CFR
Amoxicillin, every 8 h	> 10,000	.	> 10,000	.	> 10,000	.	2500	0.90
Amoxicillin-clavulanic acid, every 8 h	2100	0.91	8000	0.90	> 10,000	.	2100	0.91
Cephalexin, every 6 h	3500	0.91	4000	0.9	> 10,000	.	4000	0.9
Ciprofloxacin, every 12 h	50	0.98	700	0.9	> 10,000	.	200	0.96
Fosfomycin, every 24 h	1500	0.96	2000	0.91	3500	0.91	3000	0.9

The data presented are the lowest doses simulated for which the CFR was 0.9 or higher to the nearest 100 mg (or 50 mg if less than 100 mg). Simulations were stopped if doses exceeded 10 g without reaching a CFR 0.9

#### Amoxicillin

At a dose of 1000 mg eight hourly, CFR values were low (< 0.5) for all bacterial populations considered (≤ MIC_50_, ≤ MIC_90_, the whole Leeds population and ≤ EUCAST MIC breakpoint of 8 mg/L). For the 43% of the Leeds population at or below the EUCAST breakpoint, 2500 mg eight hourly achieved a CFR > 0.9. Standard doses of amoxicillin (1000 mg eight hourly) achieved a PTA > 0.9 for isolates with an MIC value ≤ 2 mg/L (6% of the population).

#### Amoxicillin-clavulanic acid

At 625 mg eight hourly, CFRs were < 0.9 for all bacterial populations considered (≤ MIC_50_, ≤ MIC_90_, whole Leeds population, and ≤ EUCAST MIC breakpoint of 8 mg/L). For the 62% of the Leeds *E. coli* population at or below the EUCAST breakpoint, 2250 mg of amoxicillin eight hourly achieved a CFR of > 0.9. Standard doses of amoxicillin-clavulanic acid (500 mg amoxicillin eight hourly) achieved a PTA of 0.86 for isolates with an MIC of ≤ 1 mg/L (17% of the population).

#### Cephalexin

At 1500 mg six hourly, CFRs were below 0.9 for all bacterial populations considered (≤ MIC_50_, ≤ MIC_90_, whole Leeds population, and ≤ EUCAST MIC breakpoint of 16 mg/L). For the 83% of the Leeds *E. coli* population at or below the EUCAST breakpoint, 4000 mg mg six hourly achieved a CFR > 0.9. Standard doses of cephalexin (1500 mg 8 hourly) achieved a PTA of 0.85 for isolates with an MIC of ≤ 4 mg/L (22% of the population).

#### Ciprofloxacin

At 750 mg, twelve hourly CFRs were above 0.9 when considering bacterial populations including those with MICs up to the MIC_50_, MIC_90_, and up to the EUCAST MIC breakpoint of 0.25 mg/L. For the 80% of the Leeds *E. coli* population at or below the EUCAST breakpoint, 200 mg twelve hourly achieved a CFR > 0.9. The lowest standard doses of ciprofloxacin (250 mg 12 hourly) achieved a PTA > 0.9 for isolates with an MIC up to 0.125 mg/L (76% of the population). Ciprofloxacin at 100 mg twelve hourly (a lower than standard dose) achieved a PTA > 0.9 for isolates with an MIC up to 0.06 mg/L (71% of the Leeds population).

#### Fosfomycin

At 3500 mg, 24 hourly CFRs were above 0.9 for all bacterial populations considered (≤ MIC_50_, ≤ MIC_90_, whole Leeds population, and ≤ EUCAST MIC breakpoint of 32 mg/L). For the 99% of the Leeds *E. coli* population at or below the EUCAST breakpoint, 3000 mg achieved a CFR > 0.9. The standard dose of fosfomycin (3000 mg 24 hourly) achieved a PTA > 0.9 for isolates with an MIC up to 1 mg/L (87% of the population).

## Discussion

There is clinical evidence that suggests oral antibiotics can be effective for the treatment of patients with pyelonephritis. This evidence principally relates to quinolones in the adult population, but also includes oral beta-lactams [2, 34]. In other populations, where intravenous antibiotics and quinolones are less suitable, i.e. paediatric populations, oral antibiotics including cefixime, ceftibuten, or amoxicillin-clavulanic acid are recommended in preference to intravenous antibiotics [35]. In the adult population however, there is a limited range of oral antibiotics recommended for the treatment of pyelonephritis reflecting a lack of both clinical data and PK/PD predictions to support recommendations [2]. On a background of increasing antibiotic resistance, there is a need to increase the oral antibiotic options for the treatment of pyelonephritis and PK/PD analyses can provide a basis for the identification of these alternatives.

The first, and an important finding of the study, was that there were limitations in the quality and relevance of PK models available for use in simulations. Most of the antibiotics under study have been in clinical use for many years and the PK studies do not fulfil current modelling standards. Also, some of the selected antibiotics, e.g. mecillinam, nitrofurantoin, norfloxacin, trimethoprim, are only currently recommended for use in patients with lower urinary tract infection and so, a focus may have been put on site of action data (urine) instead of blood exposure. The limitations in the PK data identified in this study are a finding of major importance. This study demonstrates the need for new pharmacokinetic studies of oral antibiotics in populations that are relevant, e.g. patients in the community with pyelonephritis, as opposed to healthy volunteers or intensive care unit patients.

The PK/PD simulation data suggest there is potential for the use of novel antibiotic dosing regimens for the oral treatment of pyelonephritis. Regarding amoxicillin, a dose of 2500 mg was predicted to be appropriate for the 43% of Leeds isolates reported as sensitive by EUCAST breakpoints. This dose is within BNF-recommended doses of amoxicillin, with a dose of 3000 mg being recommended for prophylaxis. Likewise, according to the results reported here, amoxicillin-clavulanic acid at 2250 mg of amoxicillin may have been an effective treatment for the 62% of Leeds isolates susceptible according to EUCAST. On the contrary, if the MIC_90_ or the whole of our studied bacterial population from Leeds are considered, doses higher than 10 g are needed, highlighting the need for local antibiotic susceptibility data to inform treatment recommendations. Although, the predicted effective dose of cephalexin was 4000 mg for EUCAST-susceptible isolates (above BNF-recommended dosing levels), 22% of isolates had a cephalexin MIC of ≤ 4 mg/L, an MIC associated with a high PTA at the BNF-recommended dose of 1500 mg eight hourly. These data are therefore supportive of the recommendation within the IDSA guideline to assess the role of oral broad-spectrum cephalosporins for outpatient treatment of pyelonephritis [2]. On the other hand, ciprofloxacin, an antibiotic recommended for the treatment of pyelonephritis, was predicted to be effective at standard doses; i.e., our PK/PD simulations predicted ciprofloxacin at standard doses achieved a CFR of > 0.9 at the MIC_90_. But interestingly, it was predicted that ciprofloxacin could be prescribed at lower doses whilst maintaining a PTA > 0.9, with doses as low as 100 mg being effective for most patients (71% of population). This prediction has not given consideration to mutant selection thresholds, and the selection of antibiotic resistant mutants would need to be considered in clinical trials [36]. Fosfomycin was also predicted to be clinically effective at near to standard doses (3500 mg). But this assessment is based on using a PDT of an AUC/MIC ratio of 43. This target was chosen based on published in vivo data, but in vitro data have suggested AUC/MIC targets of around 2000, which, if clinically relevant, would preclude the oral use of fosfomycin for pyelonephritis [27]. It is also known that fosfomycin develops resistance on treatment, which may again limit the drugs usefulness, though these resistant populations may have paid a biological price and be less fit, and so less clinically relevant [37].

The PTA and CFR values determined for the oral antibiotics inform the overall study aim of deciding about the potential of the investigated antibiotics to be further investigated for clinical use. The data suggests that multiple antibiotics should be further investigated. What will be needed are pharmacokinetic studies to be able to conduct simulations with more confidence. But given the conducted studies used PK data from healthy volunteers and intensive care unit patients, both groups known to have higher levels of renal excretion than other groups, it is possible that studies in relevant populations may be more favourable to assessments of antibiotic efficacies [38]. We considered performing sensitivity analysis on the models to examine this further; however, as the results of this study are hypothesis generating, we decided against this approach. Nevertheless, further work based on updated PK data should perform sensitivity analyses to explore data applicability in order to make treatment recommendations. The PK/PD simulations completed were based on a set of bacteraemia isolates from patients with pyelonephritis in Leeds. This has the advantage of being a data set that was based on a defined population, but will have inherent biases. The data are geographically and temporarily restricted, and may represent a bias to more resistant isolates being based on bacteraemia isolates from hospitalized adults as opposed to urine cultures from community patients. Indeed, compared with community *E. coli* isolates identified in urine samples from Leeds in 2010–2012, resistance rates used in this study had almost twice the rate of antibiotic resistance reported in community isolates [39]. Again, re-analysis of predicted antibiotic efficacies completed using *E. coli* isolates derived from community-based cases of pyelonephritis may be more favourable to assessments of antibiotic efficacies. Also, the simulations were conducted over the first 24 h of treatment. If simulations were extended beyond 24 h, they may also be more favourable to predictions of antibiotic efficacies.

The safety and tolerability of non-standard doses which are predicted to be clinically effective in this study simulation are not known, and so it must be clear that these non-standard doses are not treatment recommendations; i.e., they should be considered as a suggestion to reconsider standard doses until further research is performed. Oral antibiotics (ciprofloxacin and trimethoprim-sulfamethoxazole) recommended for the treatment of pyelonephritis have been associated with high rates of adverse events of 24–33% respectively [40]. It is plausible that higher than standard doses of antibiotics may result in treatments with comparable side effect profiles and tolerability.

There are a number of limitations to the presented analyses that we highlight as follows. The analyses do not account for the duration of antibiotic treatment, which might have an impact on the total exposure to the drug and therefore on the response. Moreover, the use of data obtained from critically ill patients introduces a limitation in that the model population may be very different to those receiving the hypothetical oral doses in the community. For example, the variability of pharmacokinetic parameters is likely to be greater in critically ill patients due to factors such as creatinine clearance differences in these patients, presenting a limitation that may affect the final PTA. As we decided to fix the creatinine clearance in an attempt to represent a healthy patient population, it is likely that in doing so, the overall simulated variability of the model is less than the true value. This is in addition to the limitations in selecting absorption parameters from older studies, where formulations used may be different to those currently used, as we were unable to include variance for these parameters. PK/PD targets were obtained from EUCAST rationale documents when available and their reliability impacts on the PTA results. Ideally, the targets were clinically derived; however, EUCAST use multiple sources for targets. We acknowledge therefore that the targets will vary in the accuracy of their estimates. Whilst the data presented are restricted to one patient cohort and so may limit generalisability, they allow a detailed consideration of the approaches being considered, namely the potential for novel and/or individualised dosing regimens for the oral treatment of pyelonephritis based on the susceptibility of the patient isolate, and based on geographically restricted susceptibility data. In the future, geographically restricted (local) antibiotic guidelines could be developed by inputting a specific patient population’s antibiotic susceptibilities into antibiotic simulations utilising known pharmacokinetic data.

## Conclusion

Based on the developed model quality assessment technique, limitations in the PK models available have been highlighted. Given this, there is a need for further research to develop new population PK models for antibiotics accounting for patient characteristics. Accepting the PK model limitations, the PK/PD simulations have shown that there is a rational basis for oral antibiotics including amoxicillin, amoxicillin-clavulanic acid, cephalexin, ciprofloxacin, and fosfomycin to be further investigated, both at established doses and doses above and below standard doses, for the treatment of pyelonephritis. It is also possible that novel antibiotic breakpoints should be set which would determine if an oral antibiotic could be recommended for the treatment of pyelonephritis and at which dose.

## Electronic supplementary material

ESM 1 (DOCX 104 kb)
