# Supplementary material for: A pharmacokinetic-pharmacodynamic assessment of oral antibiotics for pyelonephritis
Source: Eur J Clin Microbiol Infect Dis. 2019 Sep 7;38(12):2311–21. doi: 10.1007/s10096-019-03679-9 (PMC6858297; doi:10.1007/s10096-019-03679-9)
Supplement: Supplementary file 1 — (DOCX 104 kb) [file 10096_2019_3679_MOESM1_ESM.docx]

**Online resource**

**Article title:** A pharmacokinetic-pharmacodynamic assessment of oral antibiotics for pyelonephritis

**Journal name:** European Journal of Clinical Microbiology & Infectious Diseases

**Author names:** JWS Cattrall, E Asín-Prieto, J Freeman, IF Trocóniz, A Kirby*

**^*^Corresponding author**: Tel: +44 113 233 9239; E-mail: a.kirby@leeds.ac.uk; Address: Department of Microbiology, Old Medical School, Leeds General Infirmary, Leeds, LS1 3EX, UK

**Systematic review methods related to the identification of PK models for analysis.**

Searches were conducted for studies up to March 2017 using both MeSH terms and key word searches. The terms were combined using a mixture of both AND and OR Boolean operators to optimise output. Regarding antibiotics for which data was limited (those with less than five papers included from search: cephalexin, fosfomycin, mecillinam, nitrofurantoin, norfloxacin), secondary reference searching and manual citation tracking was then conducted on included papers to highlight further studies not previously identified in the search.

**Screening of articles**

Articles were screened using set inclusion and exclusion criteria. For inclusion, papers were required to evaluate the use of the chosen antibiotic in humans and to develop a population pharmacokinetic model based on clinical data. Papers were excluded if the antibiotic in question was administered via inhalation, the paper was unavailable in English or if the full text of a paper was not published.

Below, the search terms used as part of the systematic review for the PK analysis of the selected antibiotics are specified:

- Databases searched: Medline, EMBASE + Embase classic
- Search terms: “pharmacokinetic*”, “model”, “simulation”
- Model development software searched: NONMEM, ADAPT, Monolix, pmetrics, phoenix nlme, winbugs, jags, Kinetica, STAN, WinNonLin, MATLAB

**Results of the systematic review of articles related to the PK analysis of selected antibiotics**

The tables below include papers identified by the systematic review that were evaluated for quality. Headings describe the components of a ‘confidence in quality (CIQ)’ score. Nomenclature of relative standard error obtained from NONMEM was used (NMRSE) but other equivalent software parameter precision values were also included. NMRSE, NONMEM relative standard error; GOF, Goodness of fit (Plots) and SBMD, simulation based model diagnostics contributed to the CIQ score equally.

**Tables list**

**Systematic review tables**

**Table A1.** Quality of beta-lactams (amoxicillin, amoxicillin + clavulanic acid, cephalexin and mecillinam) studies

**Table A2.** Quality of fluoroquinolones (ciprofloxacin and norfloxacin) studies

**Table A3.** Quality of fosfomycin, nitrofurantoin and trimethoprim-based studies

**Simulation results tables**

**Table A4:** Amoxicillin simulations: PTA and CFR (**bold text**) results, administration every eight hours

**Table A5:** Amoxicillin clavulanic acid simulations: PTA and CFR (**bold text**) results, administration every eight hours

**Table A6:** Cephalexin simulations: PTA and CFR (**bold text**) results, administration every six hours

**Table A7:** Ciprofloxacin simulations: PTA and CFR (**bold text**) results, administration twice daily

**Table A8:** Fosfomycin trometamol simulations: PTA and CFR (**bold text**) results, administration once every twenty-four hours

**Included PK model assessment**

**Table A9**: Assessment of the included pharmacokinetic models in relation to desired qualities.

**Table A1.** Quality of beta-lactams (amoxicillin, amoxicillin + clavulanic acid, cephalexin and mecillinam) studies

| **Antibiotic** | **Reference** | **NMRSE** | **Bootstrap** | **GOF** | **SBMD** | **CIQ Score** |
| --- | --- | --- | --- | --- | --- | --- |
| **Amoxicillin** | **Arancibia, 1980 ^[A1]^** | No | No | No | No | 0 |
|  | **Arancibia, 1982 ^[A2]^** | No | No | No | No | 0 |
|  | **Charles, 1997 ^[A3]^** | No | No | No | No | 0 |
|  | **Dalhoff, 1982 ^[A4]^** | No | No | No | No | 0 |
|  | **Eshelman, 1978 ^[A5]^** | No | No | No | No | 0 |
|  | **Francke, 1979 ^[A6]^** | Yes | No | No | No | 1 |
|  | **Huisman de Boer, 1995 ^[A7]^** | No | No | No | No | 0 |
|  | **Isla , 2011 ^[A8]^** | No | No | No | No | 0 |
|  | **Muller, 2008 ^[A9]^** | Yes | Yes | Yes | No | 3 |
|  | **Muller, 2008 ^[A10]^** | Yes | Yes | Yes | No | 3 |
|  | **Muller, 2009 ^[A11]^** | Yes | No | No | No | 1 |
|  | **Muller, 2009 ^[A12]^** | Yes | No | Yes | No | 2 |
|  | **Paintaud, 1992 ^[A13]^** | No | No | No | No | 0 |
|  | **Piotrovskjj, 1994 ^[A14]^** | No | No | Yes | No | 1 |
|  | **Pullen, 2007 ^[A15]^** | No | No | No | No | 0 |
|  | **Spyker, 1977 ^[A16]^** | No | No | No | No | 0 |
|  | **Ullah, 2009 ^[A17]^** | No | No | No | No | 0 |
|  | **Zaid, 2010 ^[A18]^** | No | No | No | No | 0 |
| **Amoxicillin + clavulanic acid** | **Carlier, 2013 ^[A19]^** | No | Yes | Yes | Yes | 3 |
|  | **Chierakul, 2006 ^[A20]^** | No | No | No | No | 0 |
|  | **De Cock, 2015 ^[A21]^** | No | Yes | Yes | Yes | 3 |
|  | **De Velde, 2016 ^[A22]^** | No | Yes | Yes | Yes | 3 |
|  | **Fraschini, 1990 ^[A23]^** | No | No | No | No | 0 |
|  | **Grange, 1989 ^[A24]^** | No | No | No | No | 0 |
|  | **Haeseker, 2014 ^[A25]^** | No | No | Yes | No | 1 |
|  | **Landersdorfer, 2009 ^[A26]^** | No | No | Yes | Yes | 2 |
| **Cephalexin** | **Ding, 2013 ^[A27]^** | No | No | No | No | 0 |
|  | **Finkelstein, 1978 ^[A28]^** | No | No | No | No | 0 |
|  | **Greene, 1972 ^[A29]^** | No | No | Yes | No | 1 |
|  | **Greene, 1976 ^[A30]^** | No | No | No | No | 0 |
|  | **Mohamed, 2011 ^[A31]^** | No | No | No | No | 0 |
|  | **Suleiman, 1988 ^[A32]^** | No | No | No | No | 0 |
|  | **Wagner, 1977 ^[A33]^** | No | No | No | No | 0 |
| **Mecillinam** | **Bailey, 1980 ^[A34]^** | No | No | No | No | 0 |
|  | **Barriere, 1982 ^[A35]^** | No | No | No | No | 0 |
|  | **Gambertoglio, 1980 ^[A36]^** | No | No | No | No | 0 |
|  | **Meyers, 1983 ^[A37]^** | No | No | No | No | 0 |
|  | **Moukhtar, 1987 ^[A38]^** | No | No | No | No | 0 |
|  | **Neu, 1983 ^[A39]^** | No | No | No | No | 0 |
|  | **Patel, 1985 ^[A40]^** | No | No | No | No | 0 |

**Table A2.** Quality of fluoroquinolones (ciprofloxacin and norfloxacin) studies

| **Antibiotic** | **Reference** | **NMRSE** | **Bootstrap** | **GOF** | **SBMD** | | **CIQ Score** |
| --- | --- | --- | --- | --- | --- | --- | --- |
| **Ciprofloxacin** | **Breilh, 2001 ^[A41]^** | No | No | No | No | | 0 |
|  | **Cios, 2014 ^[A42]^** | No | No | Yes | No | | 1 |
|  | **Di Marco, 2004 ^[A43]^** | No | No | No | No | | 0 |
|  | **Forest, 1993 ^[A44]^** | Yes | No | Yes | No | | 2 |
|  | **Goss, 1994 ^[A45]^** | No | No | No | No | | 0 |
|  | **Khachman, 2011 ^[A46]^** | Yes | No | No | Yes | | 2 |
|  | **Landersdorfer, 2010 ^[A47]^** | No | No | No | No | | 0 |
|  | **LeBel, 1989 ^[A48]^** | No | No | No | No | | 0 |
|  | **Lettieri, 1992 ^[A49]^** | No | No | No | No | | 0 |
|  | **Martinez, 2016 ^[A50]^** | No | No | No | No | | 0 |
|  | **Meagher, 2004 ^[A51]^** | No | No | No | No | | 0 |
|  | **Miyata, 2007 ^[A52]^** | No | No | No | No | | 0 |
|  | **Montgomery, 2001 ^[A53]^** | No | No | Yes | No | | 1 |
|  | **Payen, 2003 ^[A54]^** | No | No | Yes | No | | 1 |
|  | **Pea, 2000 ^[A55]^** | No | No | No | No | | 0 |
|  | **Rajagopalan, 2003 ^[A56]^** | Yes | Yes | Yes | No | | 3 |
|  | **Roberts, 2015 ^[A57]^** | Yes | No | No | No | | 1 |
|  | **Roger, 2016 ^[A58]^** | No | No | Yes | No | | 1 |
|  | **Sadiq, 2016 ^[A59]^** | Yes | No | No | Yes | | 2 |
|  | **Sanchez Navarro, 2002 ^[A60]^** | No | No | No | No | | 0 |
|  | **Sanchez Navarro, 2002 ^[A61]^** | No | No | No | No | | 0 |
|  | **Schaefer, 1996 ^[A62]^** | Yes | No | Yes | No | | 2 |
|  | **Schuck, 2005 ^[A63]^** | No | No | No | No | | 0 |
|  | **Shah, 1994 ^[A64]^** | No | No | No | No | | 0 |
|  | **Spooner, 2011 ^[A65]^** | No | No | No | No | | 0 |
|  | **Strenkoski-Nix, 1998 ^[A66]^** | No | No | No | No | | 0 |
|  | **Thuo, 2011 ^[A67]^** | No | Yes | Yes | Yes | | 3 |
|  | **Zhao, 2015 ^[A68]^** | Yes | Yes | Yes | Yes | | 4 |
| **Norfloxacin** | **Lepage, 1991 ^[A69]^** | No | No | No | No | 0 | |
|  | **MacGowan, 1988 ^[A70]^** | No | No | No | No | 0 | |
|  | **Swanson, 1983 ^[A71]^** | No | No | No | No | 0 | |

**Table A3.** Quality of fosfomycin, nitrofurantoin and trimethoprim-based studies

| **Antibiotic** | **Reference** | **NMRSE** | **Bootstrap** | **GOF** | **SBMD** | **CIQ Score** |
| --- | --- | --- | --- | --- | --- | --- |
| **Fosfomycin** | **Frossard, 2000 ^[A72]^** | No | No | No | No | 0 |
|  | **Joukhadar, 2003 ^[A73]^** | No | No | No | No | 0 |
|  | **Parker, 2015 ^[A74]^** | No | Yes | Yes | Yes | 3 |
|  | **Rhodes, 2015 ^[A75]^** | No | No | Yes | No | 1 |
|  | **Sauermann, 2005 ^[A76]^** | No | No | Yes | No | 1 |
| **Nitrofurantoin** | **Hoener, 1981 ^[A77]^** | No | No | No | No | 0 |
|  | **Liedtke, 1980 ^[A78]^** | No | No | No | No | 0 |
|  | **Maier-Lenz, 1979 ^[A79]^** | No | No | No | No | 0 |
| **Trimethoprim and trimethoprim sulfamethoxazole** | **Alsaad, 2016 ^[A80]^** | No | Yes | Yes | Yes | 3 |
|  | **Baethke, 1972 ^[A81]^** | No | No | No | No | 0 |
|  | **Halstenson, 1984 ^[A82]^** | No | No | No | No | 0 |
|  | **Hess, 1993 ^[A83]^** | No | No | No | No | 0 |
|  | **Jeliffe, 1997 ^[A84]^** | No | No | No | No | 0 |
|  | **Lares-Asseff, 1996 ^[A85]^** | No | No | No | No | 0 |
|  | **Morgan, 1980 ^[A86]^** | No | No | No | No | 0 |
|  | **Svirbely, 1989 ^[A87]^** | No | No | No | No | 0 |

**Results of PK/PD simulations giving PTA at various doses and for various MIC values**

**Table A4:** Amoxicillin simulations: PTA and CFR (**bold text**) results, administration every eight hours

| **Proportion of Leeds isolates (%)** | **Cumulative percentage (%)** | **MIC (mg/L)** | **Dose simulated (mg)** | | | | | | | | | |  |  |
| --- | --- | --- | --- | --- | --- | --- | --- | --- | --- | --- | --- | --- | --- | --- |
|  |  |  | **250** | **500** | **750** | **1000** | **1250** | **1500** | **1750** | **2000** | **2250** | **2500** | **2750** | **3000** |
| 5.61 | 5.61 | 2 | 2 | 40.5 | 77.4 | 90.3 | 96.3 | 96.9 | 99 | 98.9 | 99.8 | 99.7 | 99.7 | 99.8 |
|  |  |  | **2** | **40.5** | **77.4** | **90.3** | **96.3** | **96.9** | **99** | **98.9** | **99.8** | **99.7** | **99.7** | **99.8** |
| 17.76 | 23.36 | 4 | 0 | 0.7 | 13.8 | 47 | 72.9 | 84.1 | 91.6 | 95.7 | 97.4 | 98.6 | 98.9 | 99 |
|  |  |  | **<1** | **10.2** | **29.1** | **57.4** | **78.6** | **87.2** | **93.4** | **96.5** | **98** | **98.9** | **99.1** | **99.2** |
| 19.63 | 42.99 | 8 | 0 | 0 | 0 | <1 | 4.3 | 18 | 36.9 | 58.2 | 72.3 | 80.4 | 88.6 | 94.5 |
|  |  |  | **<1** | **5.6** | **15.8** | **31.5** | **44.6** | **55.6** | **67.6** | **79** | **86** | **90.5** | **94.3** | **97.1** |
| 0.93 | 43.93 | 16 | 0 | 0 | 0 | 0 | 0 | 0.1 | 0.3 | 0.8 | 2.3 | 7.1 | 14.3 | 23.8 |
|  |  |  | **<1** | **5.5** | **15.4** | **30.8** | **43.7** | **54.4** | **66.1** | **77.3** | **84.4** | **88.7** | **92.6** | **95.5** |
| 0.93 | 44.86 | 32 | 0 | 0 | 0 | 0 | 0 | 0 | 0 | 0 | 0 | 0 | 0 | 0 |
|  |  |  | **<1** | **5.3** | **15.1** | **30.2** | **42.7** | **53.3** | **64.8** | **75.7** | **82.7** | **86.8** | **91** | **93.5** |
| 0.00 | 44.86 | 64 | 0 | 0 | 0 | 0 | 0 | 0 | 0 | 0 | 0 | 0 | 0 | 0 |
|  |  |  | **<1** | **5.3** | **15.1** | **30.2** | **42.7** | **53.3** | **64.8** | **75.7** | **82.7** | **86.8** | **91** | **93.5** |
| 0.00 | 44.86 | 128 | 0 | 0 | 0 | 0 | 0 | 0 | 0 | 0 | 0 | 0 | 0 | 0 |
|  |  |  | **<1** | **5.3** | **15.1** | **30.2** | **42.7** | **53.3** | **64.8** | **75.7** | **82.7** | **86.8** | **91** | **93.5** |
| 55.14 | 100.00 | 256 | 0 | 0 | 0 | 0 | 0 | 0 | 0 | 0 | 0 | 0 | 0 | 0 |
|  |  |  | **<1** | **2.4** | **6.8** | **13.6** | **19.2** | **23.9** | **29.1** | **34** | **37.1** | **38.9** | **41** | **41.9** |

**Table A5:** Amoxicillin clavulanic acid simulations: PTA and CFR (**bold text**) results, administration every eight hours

| **Proportion of Leeds isolates (%)** | **Cumulative percentage (%)** | **MIC (mg/L)** | **Dose simulated (mg of amoxicillin)** | | | | | | | | | |  |  |
| --- | --- | --- | --- | --- | --- | --- | --- | --- | --- | --- | --- | --- | --- | --- |
|  |  |  | **250** | **500** | **750** | **1000** | **1250** | **1500** | **1750** | **2000** | **2250** | **2500** | **2750** | **3000** |
| 16.98 | 16.98 | 1 | 39.3 | 86.4 | 96.4 | 97.9 | 99 | 99.3 | 99.8 | 99.9 | 100 | 99.9 | 99.9 | 100 |
|  |  |  | **39.3** | **86.4** | **96.4** | **97.9** | **99** | **99.3** | **99.8** | **99.9** | **100** | **99.9** | **99.9** | **100** |
| 20.75 | 37.74 | 2 | 0.2 | 40.5 | 77.4 | 90.3 | 96.3 | 96.9 | 99 | 98.9 | 99.8 | 99.7 | 99.7 | 99.8 |
|  |  |  | **17.8** | **61.1** | **85.9** | **93.7** | **97.5** | **98.0** | **99.3** | **99.3** | **99.9** | **99.8** | **99.8** | **99.9** |
| 10.38 | 48.11 | 4 | 0 | 0.7 | 13.8 | 47 | 72.9 | 84.1 | 91.6 | 95.7 | 97.4 | 98.6 | 98.9 | 99 |
|  |  |  | **17.8** | **61.1** | **85.9** | **93.7** | **97.5** | **98.0** | **99.3** | **99.3** | **99.9** | **99.8** | **99.8** | **99.9** |
| 14.15 | 62.26 | 8 | 0 | 0 | 0 | 0.7 | 4.3 | 18 | 36.9 | 58.2 | 72.3 | 80.4 | 88.6 | 94.5 |
|  |  |  | **17.8** | **61.1** | **85.9** | **93.7** | **97.5** | **98.0** | **99.3** | **99.3** | **99.9** | **99.8** | **99.8** | **99.9** |
| 11.32 | 73.58 | 16 | 0 | 0 | 0 | 0 | 0 | 0.1 | 0.3 | 0.8 | 2.3 | 7.1 | 14.3 | 23.8 |
|  |  |  | **17.8** | **61.1** | **85.9** | **93.7** | **97.5** | **98.0** | **99.3** | **99.3** | **99.9** | **99.8** | **99.8** | **99.9** |
| 12.26 | 85.85 | 32 | 0 | 0 | 0 | 0 | 0 | 0 | 0 | 0 | 0 | 0 | 0 | 0 |
|  |  |  | **17.8** | **61.1** | **85.9** | **93.7** | **97.5** | **98.0** | **99.3** | **99.3** | **99.9** | **99.8** | **99.8** | **99.9** |
| 3.77 | 89.62 | 64 | 0 | 0 | 0 | 0 | 0 | 0 | 0 | 0 | 0 | 0 | 0 | 0 |
|  |  |  | **17.8** | **61.1** | **85.9** | **93.7** | **97.5** | **98.0** | **99.3** | **99.3** | **99.9** | **99.8** | **99.8** | **99.9** |
| 10.38 | 100.00 | 128 | 0 | 0 | 0 | 0 | 0 | 0 | 0 | 0 | 0 | 0 | 0 | 0 |
|  |  |  | **17.8** | **61.1** | **85.9** | **93.7** | **97.5** | **98.0** | **99.3** | **99.3** | **99.9** | **99.8** | **99.8** | **99.9** |

**Table A6:** Cephalexin simulations: PTA and CFR (**bold text**) results, administration every six hours

| **Proportion of Leeds isolates (%)** | **Cumulative percentage (%)** | **MIC (mg/L)** | **Dose simulated (mg)** | | | | | | | | | | | |  |  |  |  |
| --- | --- | --- | --- | --- | --- | --- | --- | --- | --- | --- | --- | --- | --- | --- | --- | --- | --- | --- |
|  |  |  | **250** | **500** | **750** | **1000** | **1250** | **1500** | **1750** | **2000** | **2250** | **2500** | **2750** | **3000** | **3500** | **4000** | **4500** | **5000** |
| 0.94 | 0.94 | 0.5 | 91.5 | 98.3 | 99.4 | 99.9 | 99.7 | 100 | 100 | 100 | 100 | 100 | 100 | 100 | 100 | 100 | 100 | 100 |
|  |  |  | **91.5** | **98.3** | **99.4** | **99.9** | **99.7** | **100** | **100** | **100** | **100** | **100** | **100** | **100** | **100** | **100** | **100** | **100** |
| 0.00 | 0.94 | 1 | 74.4 | 91.1 | 95.8 | 98.8 | 98.9 | 99.5 | 99.7 | 99.9 | 100 | 100 | 100 | 100 | 100 | 100 | 100 | 100 |
|  |  |  | **91.5** | **98.3** | **99.4** | **99.9** | **99.7** | **100** | **100** | **100** | **100** | **100** | **100** | **100** | **100** | **100** | **100** | **100** |
| 0.00 | 0.94 | 2 | 42.7 | 73.2 | 85.4 | 92.6 | 94.5 | 96.3 | 97.6 | 98.1 | 99.1 | 99.1 | 99.7 | 99.5 | 99.6 | 99.6 | 100 | 99.9 |
|  |  |  | **91.5** | **98.3** | **99.4** | **99.9** | **99.7** | **100** | **100** | **100** | **100** | **100** | **100** | **100** | **100** | **100** | **100** | **100** |
| 20.75 | 21.70 | 4 | 16.7 | 41.7 | 62.4 | 72.1 | 77 | 84.7 | 88.7 | 90.9 | 94.5 | 95.3 | 96.1 | 97.2 | 97.8 | 98.5 | 99.1 | 99.3 |
|  |  |  | **19.9** | **44.1** | **63.9** | **73.2** | **77.9** | **85.3** | **89.2** | **91.3** | **94.7** | **95.5** | **96.2** | **97.3** | **97.9** | **98.5** | **99.1** | **99.3** |
| 44.34 | 66.04 | 8 | 3.9 | 13.4 | 29.8 | 41.8 | 48.3 | 59.3 | 68.5 | 73.7 | 74.6 | 80.2 | 84 | 86 | 87.3 | 92.3 | 95.2 | 95.2 |
|  |  |  | **9.2** | **23.5** | **41** | **52.1** | **58** | **67.8** | **75.3** | **79.5** | **81.2** | **85.2** | **88** | **89.7** | **90.8** | **94.3** | **96.5** | **96.5** |
| 16.98 | 83.02 | 16 | 0.3 | 3 | 8.9 | 17.4 | 20.3 | 27.7 | 37.5 | 43.1 | 45 | 50.1 | 58.7 | 59.9 | 64.4 | 73.7 | 80.1 | 79.5 |
|  |  |  | **7.4** | **19.3** | **34.5** | **45** | **50.3** | **59.6** | **67.6** | **72** | **73.8** | **78** | **82** | **83.6** | **85.4** | **90.1** | **93.1** | **93.1** |
| 0.94 | 83.96 | 32 | 0 | 0.3 | 1.8 | 3.6 | 6 | 8.2 | 11.9 | 14.2 | 19 | 21.8 | 28.2 | 27.4 | 33.9 | 42.6 | 47.6 | 51.2 |
|  |  |  | **7.3** | **19** | **34** | **44.6** | **49.8** | **59.1** | **66.9** | **71.4** | **73.2** | **77.4** | **81.4** | **83** | **84.8** | **89.6** | **92.6** | **92.6** |
| 4.72 | 88.68 | 64 | 0 | 0 | 0.1 | 0.2 | 0.9 | 1.3 | 2 | 2.9 | 5.1 | 5.9 | 7.2 | 8.8 | 12.1 | 18.1 | 18.2 | 21.3 |
|  |  |  | **6.9** | **18.1** | **32.3** | **42.2** | **47.2** | **56** | **63.5** | **67.7** | **69.5** | **73.6** | **77.5** | **79** | **80.9** | **85.8** | **88.6** | **88.8** |
| 3.77 | 92.45 | 128 | 0 | 0 | 0 | 0 | 0.2 | 0.3 | 0.1 | 0.4 | 0.7 | 1 | 1.1 | 1.2 | 2.9 | 4.8 | 5.4 | 5.3 |
|  |  |  | **6.6** | **17.3** | **31** | **40.5** | **45.3** | **53.7** | **60.9** | **65** | **66.8** | **70.6** | **74.4** | **75.9** | **77.8** | **82.5** | **85.3** | **85.4** |
| 7.55 | 100.00 | 256 | 0 | 0 | 0 | 0 | 0 | 0 | 0 | 0 | 0 | 0.1 | 0.1 | 0.3 | 0.1 | 0.5 | 0.8 | 1.1 |
|  |  |  | **6.1** | **16** | **28.6** | **37.4** | **41.9** | **49.7** | **56.3** | **60.1** | **61.7** | **65** | **68.8** | **70.2** | **71.9** | **76** | **79** | **79** |

**Table A7:** Ciprofloxacin simulations: PTA and CFR (**bold text**) results, administration twice daily

| **Proportion of Leeds isolates (%)** | **Cumulative percentage (%)** | **MIC (mg/L)** | **Dose simulated (mg)** | | | | | | | | | |
| --- | --- | --- | --- | --- | --- | --- | --- | --- | --- | --- | --- | --- |
|  |  |  | **100** | **200** | **300** | **400** | **500** | **600** | **700** | **800** | **900** | **1000** |
| 0.94 | 0.94 | 0.004 | 100 | 100 | 100 | 100 | 100 | 100 | 100 | 100 | 100 | 100 |
|  |  |  | **100** | **100** | **100** | **100** | **100** | **100** | **100** | **100** | **100** | **100** |
| 15.09 | 16.04 | 0.008 | 100 | 100 | 100 | 100 | 100 | 100 | 100 | 100 | 100 | 100 |
|  |  |  | **99.9** | **99.9** | **99.9** | **99.9** | **99.9** | **99.9** | **99.9** | **99.9** | **99.9** | **99.9** |
| 29.25 | 45.28 | 0.015 | 100 | 100 | 100 | 100 | 100 | 100 | 100 | 100 | 100 | 100 |
|  |  |  | **100** | **100** | **100** | **100** | **100** | **100** | **100** | **100** | **100** | **100** |
| 21.70 | 66.98 | 0.03 | 99.9 | 100 | 100 | 100 | 100 | 100 | 100 | 100 | 100 | 100 |
|  |  |  | **100** | **100** | **100** | **100** | **100** | **100** | **100** | **100** | **100** | **100** |
| 3.77 | 70.75 | 0.06 | 93.1 | 99.8 | 100 | 100 | 100 | 100 | 100 | 100 | 100 | 100 |
|  |  |  | **99.6** | **100** | **100** | **100** | **100** | **100** | **100** | **100** | **100** | **100** |
| 5.66 | 76.42 | 0.125 | 29.8 | 91.3 | 99.6 | 99.9 | 100 | 100 | 100 | 100 | 100 | 100 |
|  |  |  | **94.4** | **99.3** | **100** | **100** | **100** | **100** | **100** | **100** | **100** | **100** |
| 3.77 | 80.19 | 0.25 | 0.2 | 32.3 | 75 | 92.4 | 97.2 | 99.2 | 99.5 | 100 | 99.9 | 100 |
|  |  |  | **90.0** | **96.2** | **98.8** | **99.6** | **99.9** | **99.9** | **100** | **100** | **100** | **100** |
| 3.77 | 83.96 | 0.5 | 0 | 0 | 7.2 | 30.6 | 56 | 74.2 | 83.8 | 92.2 | 95.4 | 97.6 |
|  |  |  | **85.9** | **91.9** | **94.7** | **96.5** | **97.9** | **98.8** | **99.2** | **99.6** | **99.8** | **99.9** |
| 0.00 | 83.96 | 1 | 0 | 0 | 0 | 0.3 | 1.8 | 7.1 | 18.4 | 32.7 | 43.5 | 58.1 |
|  |  |  | **85.9** | **91.9** | **94.7** | **96.5** | **97.9** | **98.8** | **99.2** | **99.6** | **99.8** | **99.9** |
| 0.00 | 83.96 | 2 | 0 | 0 | 0 | 0 | 0 | 0 | 0 | 0.1 | 0.4 | 1.7 |
|  |  |  | **85.9** | **91.9** | **94.7** | **96.5** | **97.9** | **98.8** | **99.2** | **99.6** | **99.8** | **99.9** |
| 0.00 | 83.96 | 4 | 0 | 0 | 0 | 0 | 0 | 0 | 0 | 0 | 0 | 0 |
|  |  |  | **85.9** | **91.9** | **94.7** | **96.5** | **97.9** | **98.8** | **99.2** | **99.6** | **99.8** | **99.9** |
| 0.94 | 84.91 | 8 | 0 | 0 | 0 | 0 | 0 | 0 | 0 | 0 | 0 | 0 |
|  |  |  | **85.0** | **90.8** | **93.6** | **95.4** | **96.8** | **97.7** | **98.1** | **98.5** | **98.7** | **98.8** |
| 0.00 | 84.91 | 16 | 0 | 0 | 0 | 0 | 0 | 0 | 0 | 0 | 0 | 0 |
|  |  |  | **85.0** | **90.8** | **93.6** | **95.4** | **96.8** | **97.7** | **98.1** | **98.5** | **98.7** | **98.8** |
| 2.83 | 87.74 | 32 | 0 | 0 | 0 | 0 | 0 | 0 | 0 | 0 | 0 | 0 |
|  |  |  | **82.2** | **87.9** | **90.6** | **92.4** | **93.7** | **94.5** | **95.0** | **95.3** | **95.5** | **95.6** |
| 4.72 | 92.45 | 64 | 0 | 0 | 0 | 0 | 0 | 0 | 0 | 0 | 0 | 0 |
|  |  |  | **78.1** | **83.4** | **86.0** | **87.7** | **88.9** | **89.7** | **90.1** | **90.5** | **90.6** | **90.7** |
| 6.60 | 99.06 | 128 | 0 | 0 | 0 | 0 | 0 | 0 | 0 | 0 | 0 | 0 |
|  |  |  | **72.8** | **77.9** | **80.2** | **81.8** | **83.0** | **83.7** | **84.1** | **84.4** | **84.6** | **84.7** |
| 0.94 | 100.00 | 256 | 0 | 0 | 0 | 0 | 0 | 0 | 0 | 0 | 0 | 0 |
|  |  |  | **72.2** | **77.1** | **79.5** | **81.0** | **82.2** | **82.9** | **83.3** | **83.7** | **83.8** | **83.9** |

**Table A8:** Fosfomycin trometamol simulations: PTA and CFR (**bold text**) results, administration once every twenty-four hours

|  | **Proportion of Leeds isolates (%)** | **Cumulative percentage (%)** | **MIC (mg/L)** | **Dose simulated (mg)** | | | | | | | | | |  |
| --- | --- | --- | --- | --- | --- | --- | --- | --- | --- | --- | --- | --- | --- | --- |
|  |  |  |  | **500** | **1000** | **1500** | **2000** | **2500** | **3000** | **3500** | **4000** | **4500** | **5000** | **6000** |
|  | 0.93 | 0.93 | 0.03 | 99.9 | 100 | 100 | 100 | 100 | 100 | 100 | 100 | 100 | 100 | 100 |
|  |  |  |  | **99.9** | **100** | **100** | **100** | **100** | **100** | **100** | **100** | **100** | **100** | **100** |
|  | 0.00 | 0.93 | 0.06 | 99.3 | 100 | 100 | 100 | 100 | 100 | 100 | 100 | 100 | 100 | 100 |
|  |  |  |  | **99.9** | **100** | **100** | **100** | **100** | **100** | **100** | **100** | **100** | **100** | **100** |
|  | 10.28 | 11.21 | 0.125 | 95.9 | 99.2 | 100 | 100 | 100 | 100 | 100 | 100 | 100 | 100 | 100 |
|  |  |  |  | **96.2** | **99.3** | **100** | **100** | **100** | **100** | **100** | **100** | **100** | **100** | **100** |
|  | 13.08 | 24.30 | 0.25 | 82.5 | 96.2 | 99.1 | 99.3 | 99.6 | 99.9 | 99.9 | 99.9 | 100 | 100 | 100 |
|  |  |  |  | **88.8** | **97.6** | **99.5** | **99.6** | **99.7** | **99.9** | **99.9** | **99.9** | **100** | **100** | **100** |
|  | 41.12 | 65.42 | 0.5 | 50.1 | 83.2 | 94.3 | 96.1 | 97.4 | 98.6 | 98.9 | 98.8 | 99.8 | 99.9 | 99.8 |
|  |  |  |  | **64.5** | **88.5** | **96.2** | **97.4** | **98.3** | **99.1** | **99.3** | **99.2** | **99.9** | **99.9** | **99.9** |
|  | 21.50 | 86.92 | 1 | 7.6 | 53.5 | 73.6 | 83.4 | 89.2 | 94.3 | 95.9 | 94.4 | 97.7 | 98.7 | 98.7 |
|  |  |  |  | **50.4** | **79.9** | **90.6** | **93.9** | **96.0** | **97.9** | **98.4** | **98.0** | **99.3** | **99.6** | **99.6** |
|  | 5.61 | 92.52 | 2 | 0 | 7.4 | 33.6 | 51.9 | 64.7 | 70.2 | 78.6 | 80.3 | 86.3 | 88.5 | 92.4 |
|  |  |  |  | **47.4** | **75.5** | **87.2** | **91.4** | **94.1** | **96.2** | **97.2** | **97.0** | **98.5** | **99.0** | **99.1** |
|  | 1.87 | 94.39 | 4 | 0 | 0 | 0.6 | 7.5 | 19.7 | 29.7 | 41.5 | 49.7 | 57.5 | 64.3 | 72.1 |
|  |  |  |  | **46.4** | **74.0** | **85.5** | **89.7** | **92.7** | **94.9** | **96.1** | **96.0** | **97.7** | **98.3** | **98.6** |
|  | 0.00 | 94.39 | 8 | 0 | 0 | 0 | 0 | 0.1 | 0.5 | 2.9 | 6.9 | 11.8 | 18.3 | 30.3 |
|  |  |  |  | **46.4** | **74.0** | **85.5** | **89.7** | **92.7** | **94.9** | **96.1** | **96.0** | **97.7** | **98.3** | **98.6** |
|  | 2.80 | 97.20 | 16 | 0 | 0 | 0 | 0 | 0 | 0 | 0 | 0 | 0 | 0.2 | 1.2 |
|  |  |  |  | **45.1** | **71.9** | **83.0** | **87.1** | **90.0** | **92.2** | **93.4** | **93.2** | **94.9** | **95.4** | **95.8** |
|  | 1.87 | 99.07 | 32 | 0 | 0 | 0 | 0 | 0 | 0 | 0 | 0 | 0 | 0 | 0 |
|  |  |  |  | **44.2** | **70.5** | **81.4** | **85.5** | **88.3** | **90.4** | **91.6** | **91.5** | **93.1** | **93.6** | **94.0** |
|  | 0.00 | 99.07 | 64 | 0 | 0 | 0 | 0 | 0 | 0 | 0 | 0 | 0 | 0 | 0 |
|  |  |  |  | **44.2** | **70.5** | **81.4** | **85.5** | **88.3** | **90.4** | **91.6** | **91.5** | **93.1** | **93.6** | **94.0** |
|  | 0.00 | 99.07 | 128 | 0 | 0 | 0 | 0 | 0 | 0 | 0 | 0 | 0 | 0 | 0 |
|  |  |  |  | **44.2** | **70.5** | **81.4** | **85.5** | **88.3** | **90.4** | **91.6** | **91.5** | **93.1** | **93.6** | **94.0** |
|  | 0.00 | 99.07 | 256 | 0 | 0 | 0 | 0 | 0 | 0 | 0 | 0 | 0 | 0 | 0 |
|  |  |  |  | **44.2** | **70.5** | **81.4** | **85.5** | **88.3** | **90.4** | **91.6** | **91.5** | **93.1** | **93.6** | **94.0** |
|  | 0.93 | 100.00 | >256 | 0 | 0 | 0 | 0 | 0 | 0 | 0 | 0 | 0 | 0 | 0 |
|  |  |  |  | **43.8** | **69.8** | **80.7** | **84.7** | **87.5** | **89.6** | **90.7** | **90.6** | **92.2** | **92.8** | **93.1** |

**Table A9**: Assessment of the included pharmacokinetic models in relation to desired qualities.

|  |  | **Population of patients with pyelonephritis** | **Predominantly female population** | **Oral admin. of antibiotic** | **HPLC drug concentration assay** | **Minimum of 60 patients** | **Multiple diagnostic checks** |
| --- | --- | --- | --- | --- | --- | --- | --- |
| Amoxicillin,  Amoxicillin-clavulanic acid | De Velde **^[^**^A22^**^]^** (2016) | No | No | Yes | Yes | No | Yes |
| Cephalexin | Greene **^[^**^A29^**^]^** (1972) | No | No | No | No | No | No |
| Ciprofloxacin | Khachman **^[^**^A46^**^]^** (2011) | No | No | No | Yes | Yes | Yes |
| Fosfomycin | Parker **^[^**^A74^**^]^** (2015) | No | No | No | Yes | No | Yes |

**Example simulation coding**

In order to perform PK/PD analysis and Monte Carlo simulation, the models from the literature were implemented computationally using MlxTRAN language (see the following example for amoxicillin PK model).

**Amoxicillin**

**Paper:** De Velde [A22]

Amoxicillin Model Simulx Script, De Velde

'AmoxicillinModelSimulxScript.txt'

[LONGITUDINAL]

input = {F, N, MTT, Vm, Km, Vc, CL, Vp, Q}

PK:

depot(target=Atr, p=F)

EQUATION:

Ktr=(N+1)/MTT

ddt_Atr = -Ktr*Atr

ddt_Ag = Ktr*Atr-((Vm*Ag)/(Km+Ag))

ddt_Ac = ((Vm*Ag)/(Km+Ag))-((Q/Vc)*Ac)+((Q/Vp)*Ap)-((CL/Vc)*Ac)
ddt_Ap = ((Q/Vc)*Ac)-((Q/Vp)*Ap)

Cc=Ac/Vc

[INDIVIDUAL]

input = {CL_pop, omega_CL, Vc_pop, omega_Vc, MTT_pop, omega_MTT, N_pop, omega_N, Vm_pop, omega_Vm, Km_pop, omega_Km}

DEFINITION:

CL = {distribution = lognormal, reference = CL_pop, var = omega_CL}

Vc = {distribution = lognormal, reference = Vc_pop, var = omega_Vc}

MTT = {distribution = lognormal, reference = MTT_pop, var = omega_MTT}

N = {distribution = lognormal, reference = N_pop, var = omega_N}

Vm = {distribution = lognormal, reference = Vm_pop, var = omega_Vm}

Km = {distribution = lognormal, reference = Km_pop, var = omega_Km}

Afterwards, the implemented PK model is used as input for the simulations. Following is the R script used for the simulations and estimation of the PTA and CFR in the example of amoxicillin.

*########### Setting the working directory and loading mlxR package.*

setwd("~/Simulations/Amoxicillin Simulations")

library(mlxR)

*########### Inputs regarding administration schedule (adm), output of the simulation (Cc), ########### parameter values (p) and level of variability and virtual population size (g)*

adm <- list(time = c(0, 8, 16), amount = 250)

Cc <- list(name ='Cc', time=seq(from=0, to=24, by=0.01))

p <- c(F= 0.70, N_pop= 4.41, omega_N= 1.2769, MTT_pop= 0.524, omega_MTT= 0.219024,

Vm_pop = 1220, omega_Vm = 0.101761, Km_pop= 287,

omega_Km= 8.3521, CL_pop = 21.3, omega_CL = 0.066564,

Vc_pop= 27.7, omega_Vc= 0.118336 ,Vp= 3.02, Q= 1.7)

g <- list(size=1000, level='individual')

*########### Command to perform the simulations. The results will be stored in the object “res”*

res <- exposure(model='AmoxicillinModelSimulxScript.txt', parameter=p, output=Cc, treatment=adm, group=g)

*########### Estimation of the probability of success of the treatment using as input the results of ########### the simulation (res), the pharmacodynamic target (PDT), the protein binding (PB), the ########### MIC data (MIC) and the inter-dose interval (II)*

TabMIC(res,PDT=32.5,PB=0.2, MIC=c(0.125, 0.25, 0.5, 1, 2, 4, 8, 16, 32, 64, 128, 256), II=8)

*########### The output is a vector with the PTA at each MIC value.*

*########### “TabMIC()” is a function that calculates the time during which the unbound ########### concentrations of the drug remain above the MIC. Developed by Eduardo Asin-Prieto.*

**Online resource references**

[A1] Arancibia A, Guttmann J, Gonzalez C. Absorption and disposition kinetics of amoxicillin in normal human subjects. *Antimicrob Agents Chemother*. 1980; 17(2): 199–202.

[A2] Arancibia A, Droguett MT, Fuentes G. Pharmacokinetics of amoxicillin in subjects with normal and impaired renal function. *Int J Clin Pharmacol Ther Toxicol.* 1982; 20(10): 447–A53.

[A3] Charles BG, Preechagoon Y, Lee TC, Steer PA, Flenady VJ, Debuse N. Population pharmacokinetics of intravenous amoxicillin in very low birth weight infants. *J Pharm Sci*. 1997; 86(11): 1288–92.

[A4] Dalhoff A, Koeppe P. Comparative pharmacokinetic analysis of amoxycillin using open two and three-compartment models. *Eur J Clin Pharmacol.* 1982; 22(3): 273–9.

[A5] Eshelman FN, Spyker DA. Pharmacokinetics of amoxicillin and ampicillin: crossover study of the effect of food. *Antimicrob Agents Chemother*. 1978; 14(4): 539–43.

[A6] Francke EL, Appel GB, Neu HC. Kinetics of intravenous amoxicillin in patients on long-term dialysis. *Clin Pharmacol Ther*. 1979; 26(1): 31–5.

[A7] Huisman-de Boer JJ, van den Anker JN, Vogel M, Goessens WH, Schoemaker RC, de Groot R. Amoxicillin pharmacokinetics in preterm infants with gestational ages of less than 32 weeks. *Antimicrob Agents Chemother.* 1995; 39(2): 431–4.

[A8] Isla A, Troconiz IF, Canut A, Labora A, Martin-Herrero JE, Pedraz JL, et al. Pharmacokinetic/pharmacodynamic evaluation of amoxicillin, amoxicillin/clavulanate and ceftriaxone in the treatment of paediatric acute otitis media in Spain. *Enferm Infecc Microbiol Clin*. 2011; 29(3): 167–73.

[A9] Muller AE, DeJongh J, Oostvogel PM, Voskuyl RA, Dörr PJ, Danhof M, et al. Amoxicillin pharmacokinetics in pregnant women with preterm premature rupture of the membranes. *Am J Obstet Gynecol*. 2008; 198(1): 108.e1-108.e6.

[A10] Muller AE, Dorr PJ, Mouton JW, De Jongh J, Oostvogel PM, Steegers EA, et al. The influence of labour on the pharmacokinetics of intravenously administered amoxicillin in pregnant women. *Br J Clin Pharmacol.* 2008; 66(6): 866–74.

[A11] Muller AE, Liefaard L, Dorr PJ, Oostvogel PM, Dejongh J, Steegers EAP, et al. Evaluation of dosing regimen on amoxicillin exposure in pregnant women with preterm premature rupture of the membranes using Monte Carlo simulation. *Int J Gen Mol Microbiol.* 2009; 95: 83–4.

[A12] Muller AE, Oostvogel PM, DeJongh J, Mouton JW, Steegers EA, Dorr PJ, et al. Pharmacokinetics of amoxicillin in maternal, umbilical cord, and neonatal sera. *Antimicrob Agents Chemother*. 2009; 53(4): 1574–80.

[A13] Paintaud G, Alvan G, Dahl ML, Grahnen A, Sjovall J, Svensson JO. Nonlinearity of amoxicillin absorption kinetics in human. *Eur J Clin Pharmacol*. 1992; 43(3): 283–8.

[A14] Piotrovskij VK, Paintaud G, Alvan G, Trnovec T. Modeling of the saturable time-constrained amoxicillin absorption in humans. *Pharm Res*. 1994; 11(9): 1346–51.

[A15] Pullen J, Driessen M, Stolk LM, Degraeuwe PL, van Tiel FH, Neef C, et al. Amoxicillin pharmacokinetics in (preterm) infants aged 10 to 52 days: effect of postnatal age. *Ther Drug Monit.* 2007; 29(3): 376–80.

[A16] Spyker DA, Rugloski RJ, Vann RL, O’Brien WM. Pharmacokinetics of amoxicillin: dose dependence after intravenous, oral, and intramuscular administration. *Antimicrob Agents Chemother.* 1977; 11(1): 132–41.

[A17] Ullah Md A, Azad MAK, Sultana R, Kabir ER, Latif AHMM, Hasnat A. Pharmacokinetic study of amoxicillin capsule in healthy Bangladeshi subjects using urinary excretion data. *Dhaka Univ J Pharm Sci*. 2009; 8(1): 53–9.

[A18] Zaid AN, Cortesi R, Kort J, Sweileh W. Interchangeability of two 500 mg amoxicillin capsules with one 1000 mg amoxicillin tablet after a single oral administration. *Indian J Pharm Sci*. 2010; 72(4): 414–20.

[A19] Carlier M, Noe M, De Waele JJ, Stove V, Verstraete AG, Lipman J, et al. Population pharmacokinetics and dosing simulations of amoxicillin/clavulanic acid in critically ill patients. *J Antimicrob Chemother*. 2013; 68(11): 2600–8.

[A20] Chierakul W, Wangboonskul J, Singtoroj T, Pongtavornpinyo W, Short JM, Maharjan B, et al. Pharmacokinetic and pharmacodynamic assessment of co-amoxiclav in the treatment of melioidosis. *J Antimicrob Chemother*. 2006; 58(6): 1215–20.

[A21] De Cock PAJG, Standing JF, Barker CIS, De Jaeger A, Dhont E, Carlier M, et al. Augmented renal clearance implies a need for increased amoxicillin-clavulanic acid dosing in critically ill children. *Antimicrob Agents Chemother.* 2015; 59(11): 7027–35.

[A22] De Velde F, de Winter BCM, Koch BCP, van Gelder T, Mouton JW. Non-linear absorption pharmacokinetics of amoxicillin: Consequences for dosing regimens and clinical breakpoints. *J Antimicrob Chemother*. 2016; 71(10): 2909–17.

[A23] Fraschini F, Scaglione F, Falchi M, Dugnani S, Mezzetti M, Cicchetti F, et al. Pharmacokinetics and tissue distribution of amoxicillin plus clavulanic acid after oral administration in man. *J Chemother.* 1990; 2(3): 171–7.

[A24] Grange JD, Gouyette A, Gutmann L, Amiot X, Kitzis MD, Islam S, et al. Pharmacokinetics of amoxycillin/clavulanic acid in serum and ascitic fluid in cirrhotic patients. *J Antimicrob Chemother*. 1989; 23(4): 605–11.

[A25] Haeseker M, Havenith T, Stolk L, Neef C, Bruggeman C, Verbon A. Is the standard dose of amoxicillin-clavulanic acid sufficient? *BMC Pharmacol Toxicol*. 2014; 15(1).

[A26] Landersdorfer CB, Kinzig M, Bulitta JB, Hennig FF, Holzgrabe U, Sorgel F, et al. Bone penetration of amoxicillin and clavulanic acid evaluated by population pharmacokinetics and Monte Carlo simulation. *Antimicrob Agents Chemother*. 2009; 53(6): 2569–78.

[A27] Yi Ding WenXing Liu, Yan Yan Jia, ChengTao Lu, Xin Jin, Jing Yang, YanRong Zhu, Lin Yang, Ying Song, LiKun Ding and AW. Effects of Amlodipine on the Oral Bioavailability of Cephalexin and Cefuroxime Axetil in Healthy Volunteers. *J Clin Pharmacol*. 2012; 53(1): 82–6.

[A28] Finkelstein E, Quintiliani R, Lee R, Bracci A, Nightingale CH. Pharmacokinetics of Oral Cephalosporins: Cephradine and Cephalexin. *J Pharm Sci*. 1978; 67(10): 1447–50.

[A29] Greene DS, Quintiliani R, Nightingale CH. Physiological Perfusion Model for Cephalosporin Antibiotics I: Model Selection Based on Blood Drug Concentrations Table I-Plasma Flows and Organ Weights for Perfusion Model Calculations. *Eur* *J Clin Pharmacol*. 1972; 61(2): 191–6.

[A30] Greene DS, Flanagan DR, Quintiliani R, Nightingale CH. Pharmacokinetics of cephalexin: an evaluation of one- and two compartment model pharmacokinetics. *J Clin Pharmacol*. 1976; 16(6): 257–64.

[A31] Mohamed SS, Mustafa MA, Ahmed EA, Algarai NA, Alawad ZA, Ali AA. Comparative Pharmacokinetics and Bioequivalence Studies of Three Oral Cephalexin Monohydrate Formulations. *Jordan J Pharm Sci*. 2011; 4(2).

[A32] Suleiman MS, Najib NM, El-Sayed YM, Abdulhameed ME. A Bioequivalence Study of Six Brands of Cephalexin. *J Clin Pharm Ther*. 1988; 13(1): 65–72.

[A33] Wagner JG. Pharmacokinetic Parameters Estimated from Intravenous Data by Uniform Methods and Some of Their Uses. *J Pharmacokinet Biopharm*. 1977; 5(2).

[A34] Bailey K, Cruickshank J, Bisson P, Radford B. Mecillinam in patients on haemodialysis. Br *J Clin Pharmacol*. 1980; 10(2): 177–80.

[A35] Barriere SL, Gambertoglio JG, Lin ET, Conte JE. Multiple-Dose Pharmacokinetics of Amdinocillin in Healthy Volunteers. *Antimicrob Agents Chemother.* 1982; 21(1): 54–7.

[A36] Gambertoglio JG, Barriere SL, Lin ET, Conte JR JE. Pharmacokinetics of Mecillinam in Healthy Subjects. *Drug Metabol Drug Interact.* 1990; 8(2): 149–58.

[A37] Meyers BR, Jacobson J, Masci J, Srulevitch E, Hirschman SZ. Pharmacokinetics of amdinocillin in healthy adults. *Antimicrob Agents Chemother*. 1983; 23(6): 827–30.

[A38] Moukhtar I, Nawishy S, Sabbour M. Pharmacokinetics of mecillinam after a single intravenous dose in patients with impaired renal function. *Int* *J Clin Pharmacol* *Res*. 1987; 7(1): 59–62.

[A39] Neu HC. Pharmacokinetics of Amdinocillin and Pivamdinocillin in Normal Volunteers. *Am J Med*. 1983; 75(2A): 60–4.

[A40] Patel I, Bornemann L, Brocks V, Fang L, Tolkoff-Rubin N, Rubin R. Pharmacokinetics of intravenous amdinocillin in healthy subjects and patients with renal insufficiency. *Antimicrob Agents Chemother*. 1985; 28(1): 46–50.

[A41] Breilh D, Saux MC, Maire P, Vergnaud JM, R WJ. Mixed pharmacokinetic population study and diffusion model to describe ciprofloxacin lung concentrations. *Comput Biol Med.* 2001; 31(3): 147–55.

[A42] Cios A, Wyska E, Szymura-Oleksiak J, Grodzicki T. Population pharmacokinetic analysis of ciprofloxacin in the elderly patients with lower respiratory tract infections. *Exp Gerontol.* 2014; 57: 107–13.

[A43] Di Marco MP, Chen J, Wainer IW, Ducharme MP. A population pharmacokinetic-metabolism model for individualizing ciprofloxacin therapy in ophthalmology. *Ther Drug Monit.* 2004; 26(4): 401–7.

[A44] Forrest A, Ballow CH, Nix DE, Birmingham MC, Schentag JJ. Development of a population pharmacokinetic model and optimal sampling strategies for intravenous ciprofloxacin. *Antimicrob Agents Chemother*. 1993; 37(5): 1065–72.

[A45] Goss TF, Forrest A, Nix DE, Ballow CH, Birmingham MC, Cumbo TJ, et al. Mathematical examination of dual individualization principles (II): The rate of bacterial eradication at the same area under the inhibitory curve is more rapid for ciprofloxacin than for cefmenoxime. *Ann Pharmacother.* 1994; 28(8): 863–8.

[A46] Khachman D, Conil J, Georges B, Saivin S, Houin G, Toutain P, et al. Optimizing ciprofloxacin dosing in intensive care unit patients through the use of population pharmacokinetic-pharmacodynamic analysis and monte carlo simulations. *J Antimicrob Chemother*. 2011; 66(8): 1798–809.

[A47] Landersdorfer CB, Kirkpatrick CM, Kinzig M, Bulitta JB, Holzgrabe U, Jaehde U, et al. Competitive inhibition of renal tubular secretion of ciprofloxacin and metabolite by probenecid. Br *J Clin Pharmacol*. 2010; 69(2): 167–78.

[A48] LeBel M, Barbeau G, Bergeron M, Roy D, Vallée F. Pharmacokinetics of ciprofloxacin in elderly patients. *Arzneimittelforschung*. 1989; 39: 523–7.

[A49] Lettieri J, Rogge M, Kaiser L, Echols R, Heller A. Pharmacokinetic profile of ciprofloxacin after single intravenous and oral doses. *Antimicrob Agents Chemother*. 1992; 36(5): 993–6.

[A50] Martinez M, Mistry B, Lukacova V, Polli J, Hoag S, Dowling T, et al. Use of Modeling and Simulation Tools for Understanding the Impact of Formulation on the Absorption of a Low Solubility Compound: Ciprofloxacin. *AAPS J*. 2016; 18(4): 886–97.

[A51] Meagher AK, Forrest A, Dalhoff A, Stass H, Schentag JJ. Novel pharmacokinetic-pharmacodynamic model for prediction of outcomes with an extended-release formulation of ciprofloxacin. *Antimicrob Agents Chemother*. 2004; 48(6): 2061–8.

[A52] Miyata K, Ohtani H, Tsujimoto M, Sawada Y. Antacid interaction with new quinolones: Dose regimen recommendations based on pharmacokinetic, model of clinical data for ciprofloxacin, gatifloxacin and norfloxacin and metal cations. *Int* *J Clin Pharmacol* Ther. 2007; 45(1): 63–70.

[A53] Montgomery MJ, Beringer PM, Aminimanizani A, Louie SG, Shapiro BJ, Jelliffe R, et al. Population pharmacokinetics and use of Monte Carlo simulation to evaluate currently recommended dosing regimens of ciprofloxacin in adult patients with cystic fibrosis. *Antimicrob Agents Chemother*. 2001; 45(12): 3468–73.

[A54] Payen S, Serreau R, Munck A, Aujard Y, Aigrain Y, Bressolle F, et al. Population Pharmacokinetics of Ciprofloxacin in Pediatric and Adolescent Patients with Acute Infections. *Antimicrob Agents Chemother*. 2003; 47(10): 3170–8.

[A55] Pea F, Milaneschi R, Baraldo M, Lugatti E, Talmassons G, Furlanut M. Ciprofloxacin disposition in elderly patients with LRTI being treated with sequential therapy (200 mg intravenously twice daily followed by 500 mg per os twice daily): comparative pharmacokinetics and the role of therapeutic drug monitoring. *Ther Drug Monit*. 2000; 22(4): 386–91.

[A56] Rajagopalan P, Gastonguay MR. Population pharmacokinetics of ciprofloxacin in pediatric patients. *J Clin Pharmacol*. 2003; 43(7): 698–710.

[A57] Roberts DM, Liu X, Roberts JA, Nair P, Cole L, Roberts MS, et al. A multicenter study on the effect of continuous hemodiafiltration intensity on antibiotic pharmacokinetics. *Crit Care*. 2015; 19(1): 84.

[A58] Roger C, Wallis SC, Louart B, Lefrant JY, Lipman J, Muller L, et al. Comparison of equal doses of continuous venovenous haemofiltration and haemodiafiltration on ciprofloxacin population pharmacokinetics in critically ill patients. *J Antimicrob Chemother*. 2016; 71(6): 1643–50.

[A59] Sadiq MW, Nielsen EI, Khachman D, Conil JM, Georges B, Houin G, et al. A whole-body physiologically based pharmacokinetic (WB-PBPK) model of ciprofloxacin: a step towards predicting bacterial killing at sites of infection. *J Pharmacokinet Pharmacodyn*. 2016; 44(2): 69-79.

[A60] Sánchez Navarro MD, Coloma Milano C, Zarzuelo Castañeda A, Sayalero Marinero ML, Sánchez-Navarro A. Pharmacokinetics of ciprofloxacin as a tool to optimise dosage schedules in community patients. *Clin Pharmacokinet*. 2002; 41(14): 1213–20.

[A61] Sanchez Navarro MD, Sayalero Marinero ML, Sanchez Navarro A. Pharmacokinetic/pharmacodynamic modelling of ciprofloxacin 250 mg/12 h versus 500 mg/24 h for urinary infections. *J Antimicrob Chemother*. 2002; 50(1): 67–72.

[A62] Schaefer HG, Stass H, Wedgwood J, Hampel B, Fischer C, Kuhlmann J, et al. Pharmacokinetics of ciprofloxacin in pediatric cystic fibrosis patients. *Antimicrob Agents Chemother*. 1996; 40(1): 29–34.

[A63] Schuck EL, Dalhoff A, Stass H, Derendorf H. Pharmacokinetic/pharmacodynamic (PK/PD) evaluation of a once-daily treatment using ciprofloxacin in an extended-release dosage form. Infection. 2005; 33(SUPPL2): 22–8.

[A64] Shah A, Lettieri J, Kaiser L, Echols R, Heller AH. Comparative pharmacokinetics and safety of ciprofloxacin 400 mg iv thrice daily versus 750 mg po twice daily. *J Antimicrob Chemother*. 1994; 33(4): 795–801.

[A65] Spooner AM, Deegan C, D’Arcy DM, Gowing CM, Donnelly MB, Corrigan OI. An evaluation of ciprofloxacin pharmacokinetics in critically ill patients undergoing continuous veno-venous haemodiafiltration. *BMC Clin Pharmacol*. 2011; 11(1): 11.

[A66] Strenkoski-Nix LC, Forrest A, Schentag JJ, Nix DE. Pharmacodynamic interactions of ciprofloxacin, piperacillin, and piperacillin/tazobactam in healthy volunteers. *J Clin Pharmacol*. 1998; 38(11): 1063–71.

[A67] Thuo N, Ungphakorn W, Karisa J, Muchohi S, Muturi A, Kokwaro G, et al. Dosing regimens of oral ciprofloxacin for children with severe malnutrition: A population pharmacokinetic study with Monte Carlo simulation. *J Antimicrob Chemother*. 2011; 66(10): 2336–45.

[A68] Zhao W, Hill H, Le Guellec C, Neal T, Mahoney S, Paulus S, et al. Population pharmacokinetics of ciprofloxacin in neonates and young infants less than three months of age. *Antimicrob Agents Chemother*. 2015; 58(11): 6572-80.

[A69] Lepage JY, Caillon J, Malinowsky JM, Lequerre S, Cozian A, Normand Y Le, et al. Pharmacokinetics of norfloxacin in the elderly. *Fundam Clin Pharmacol.* 1991; 5(3): 203–8.

[A70] Macgowan AP, Greig MA, Clarke EA, White LO, Reeves DS. The pharmacokinetics of norfloxacin in the aged. *J Antimicrob Chemother*. 1988; 22(5): 721–7.

[A71] Swanson BN, Boppana VK, Vlasses PH, Rotmensch HH, Ferguson RK. Norfloxacin disposition after sequentially increasing oral doses. *Antimicrob Agents Chemother*. 1983; 23(2): 284–8.

[A72] Frossard M, Joukhadar C, Erovic BM, Dittrich P, Mrass PE, Van Houte M, et al. Distribution and antimicrobial activity of fosfomycin in the interstitial fluid of human soft tissues. *Antimicrob Agents Chemother*. 2000; 44(10): 2728-32.

[A73] Joukhadar C, Klein N, Dittrich P, Zeitlinger M, Geppert A, Skhirtladze K, et al. Target site penetration of fosfomycin in critically ill patients. *J Antimicrob Chemother*. 2003; 51(5): 1247-52.

[A74] Parker SL, Frantzeskaki F, Wallis SC, Diakaki C, Giamarellou H, Koulenti D, et al. Population pharmacokinetics of fosfomycin in critically ill patients. *Antimicrob Agents Chemother*. 2015; 59(10): 6471-6476.

[A75] Rhodes NJ, Gardiner BJ, Neely MN, Grayson ML, Ellis AG, Lawrentschuk N, et al. Optimal timing of oral fosfomycin administration for pre-prostate biopsy prophylaxis. *J Antimicrob Chemother*. 2014; 70(7): 2068-73.

[A76] Sauermann R, Karch R, Langenberger H, Kettenbach J, Mayer-Helm B, Petsch M, et al. Antibiotic abscess penetration: Fosfomycin levels measured in pus and simulated concentration-time profiles. *Antimicrob Agents Chemother*. 2005; 49(11): 4448-4454.

[A77] Hoener B-A, Patterson SE, San F. Nitrofurantoin disposition. *Clin Pharmacol Ther.* 1981; 29(6): 808-16.

[A78] Liedtke RK, Ebel S, Missler B, Haase W, Stein L. Single-dose pharmacokinetics of macrocrystalline nitrofurantoin formulations. *Arzneimittel-Forschung.* 1980; 30(5): 833—836.

[A79] Maier-Lenz H, Ringwelski L, Windorfer A. Comparative pharmacokinetics and relative bioavailability for different preparations of nitrofurantoin. *Arzneimittel-Forschung.* 1979; 29(12): 1898–901.

[A80] Alsaad N, Dijkstra JA, Akkerman OW, De Lange WCM, Van Soolingen D, Kosterink JGW, et al. Pharmacokinetic evaluation of sulfamethoxazole at 800 milligrams once daily in the treatment of tuberculosis. *Antimicrob Agents Chemother*. 2016; 60(7): 3942–7.

[A81] Baethke R, Golde G, Gahl G. Sulphamethoxazole/trimethoprim: Pharmacokinetic studies in patients with chronic renal failure. *Eur* *J Clin Pharmacol*. 1972; 4(4): 233–40.

[A82] Halstenson CE, Blevins RB, Salem NG, Matzke GR. Trimethoprim-sulfamethoxazole pharmacokinetics during continuous ambulatory peritoneal dialysis. *Clin Nephrol.* 1984; 22(5): 239–43.

[A83] Hess MM, Boucher BA, Laizure SC, Stevens RC, Sanders PL, Janning SW, et al. Trimethoprim-sulfamethoxazole pharmacokinetics in trauma patients. *Pharmacotherapy*. 1993; 13(6): 602–6.

[A84] Jelliffe RW, Gomis P, Tahani B, Ruskin J, Sattler FR. A population pharmacokinetic model of trimethoprim in patients with pneumocystis pneumonia, made with parametric and nonparametric methods. *Ther Drug Monit*. 1997; 19(4): 450–9.

[A85] Lares-Asseff I, Villegas F, Perez G, Toledo A, Camacho A, Lopez DC. Kinetic effects of trimethoprim-sulfamethoxazole in children with biliary atresia: a new dosing regimen. *Arch Med Res.* 1996; 27(2): 183–90.

[A86] Morgan DJ, Raymond K. Evaluation of slow infusions of Co-Trimoxazole by using predictive pharmacokinetics. *Antimicrob Agents Chemother*. 1980; 17(2): 132–7.

[A87] Svirbely JE, Pesce AJ, Singh S, O’Flaherty EJ. Co-trimoxazole (sulphamethoxazole plus trimethoprim) peritoneal barrier transfer pharmacokinetics. *Clin Pharmacokinet.* 1989; 16(5): 317–25.
